# Effect of bile reflux on gastric juice microbiota in patients with different histology phenotypes

**DOI:** 10.1186/s13099-024-00619-7

**Published:** 2024-05-07

**Authors:** Yong Sung Kim, Tatsuya Unno, Seon-Young Park, Jin Ook Chung, Yoo-Duk Choi, Su-Mi Lee, Seong Hyun Cho, Dong Hyun Kim, Hyun-Soo Kim, Young Do Jung

**Affiliations:** 1 Digestive Disease Research Institute, Wonkwang University School of Medicine, Iksan, South Korea; 2 Department of Biological Sciences and Biotechnology, Chungbuk National University, Seowon-Gu, Cheongju, 28644 South Korea; 3 Division of Gastroenterology, Department of Internal Medicine, Chonnam National University Medical School, 42 Jaebong-ro, Donggu, Gwangju, 61572 South Korea; 4 Division of Endocrinology, Department of Internal Medicine, Chonnam National University Medical School, Gwangju, South Korea; 5 Department of Pathology, Chonnam National University Medical School, Gwangju, South Korea; 6 Department of Biochemistry, Chonnam National University Medical School, Gwangju, South Korea

## Abstract

**Background/aims:**

Bile reflux (BR) can influence the gastric environment by altering gastric acidity and possibly the gastric microbiota composition. This study investigated the correlation between bile acids and microbial compositions in the gastric juice of 50 subjects with differing gastric pathologies.

**Methods:**

This study included 50 subjects, which were categorized into three groups based on the endoscopic BR grading system. The primary and secondary bile acid concentrations in gastric juice samples were measured, and microbiota profiling was conducted using 16 S rRNA gene sequencing.

**Results:**

Significant differences were observed in each bile acid level in the three endoscopic BR groups (*P* < 0.05). The Shannon index demonstrated a significant decrease in the higher BR groups (*P* < 0.05). Analysis of the β-diversity revealed that BR significantly altered the gastric microbiota composition. The presence of neoplastic lesions and the presence of *H. pylori* infection impacted the β-diversity of the gastric juice microbiota. The abundance of the *Streptococcus* and *Lancefielfdella* genera exhibited positive correlations for almost all bile acid components(*P* < 0.05). In addition, the abundance of *Slobacterium*, *Veillonella*, and *Schaalia* showed positive correlations with primary unconjugated bile acids (*P* < 0.05).

**Conclusion:**

Changes in microbial diversity in the gastric juice were associated with BR presence in the stomach. This result suggests that the degree of BR should be considered when studying the gastric juice microbiome.

**Supplementary Information:**

The online version contains supplementary material available at 10.1186/s13099-024-00619-7.

## Background

Bile reflux (BR) refers to the condition whereby duodenal contents, such as bile and pancreatic juice, are refluxed back into the stomach [1]. Recent studies have shown that BR is associated with esophageal and gastric carcinogenesis and is linked to premalignant upper gastrointestinal (GI) lesions, such as Barrett’s esophagus, atrophic gastritis, and intestinal metaplasia [1–5]. Bile acid in the stomach can disrupt the mucosal barrier by dissolving the phospholipid layer in the epithelial membrane, inhibiting nitric oxide enzymes and the sodium–hydrogen exchanges in cells, stimulating histamine release from mast cells, and promoting the reverse diffusion of hydrogen ions. These changes can lead to intracellular DNA damage, apoptosis, mutation, and chronic inflammation of gastric mucosa [6]. Recent research has focused on dysbiosis in upper GI diseases; however, the gastric microbiota composition is dynamic and influenced by various factors, including diets, medication use, inflammation of gastric mucosa, and the presence of *Helicobacter pylori* (*H. pylori*) [7]. BR may also affect gastric acidity, which could significantly impact a microbial community in the stomach. Moreover, most studies investigating gastric dysbiosis have relied on mucosal biopsy samples, with few studies utilizing gastric fluid samples [8, 9]. Additionally, the relationship between intragastric bile and the microbiota in gastric juice remains poorly understood. Therefore, this study aimed to explore the association between bile acids and the microbiota in gastric juice in subjects with various gastric pathologies.

## Methods

### Subjects

A total of 50 subjects were included in this study. Subjects with a prior history of gastric surgery, any malignancy, and those taking medications, such as steroids, prokinetics, lipid-lowering agents, bile acid sequestrants, ursodeoxycholic acid (UDCA), and chenodeoxycholic acid (CDCA), were excluded from the study. Before any endoscopic procedures were conducted, the patients were provided with comprehensive information about the study, and informed consent was obtained from all subjects. All methods were performed in accordance with the Declaration of Helsinki. This study was approved by our institutional review board (Chonnam National University Hospital Institutional Review Board, IRB No. CNUH-2020-085). Written informed consent was obtained from all individuals included in the publication of this manuscript and any accompanying figures.

### Collection of gastric juice

In this study, all endoscopic procedures were performed by an experienced endoscopist (SYP) without using antifoaming and mucolytic agents or antispasmodics. Before the procedure, subjects were asked to fast for 12 h, and the endoscopy was performed as the first appointment in the early morning. The endoscope was inserted into the stomach, and the amount of bile acid in the gastric fluid was evaluated at the fundus and greater curvature of the gastric body. Since some subjects had little gastric fluid, 20 mL of distilled water was injected into the stomach and mixed with the gastric fluid in each subject. Then, the mixed gastric fluid was aspirated via the endoscopic aspiration channel using a sterile collection trap. The collected fluid specimens were immediately cryopreserved at -80 °C.

### Measurement of bile acids

#### Gross measurement by endoscopic grading

In this study, the endoscopic BR grade was defined as follows: grade 0: no bile reflux; grade 1: light-yellow-clear fluid in the stomach; grade 2: yellowish-green fluid in the stomach (Fig. S1).

#### Laboratory measurement using liquid chromatography with tandem mass spectrometry

The bile acid profiles of the aspirated gastric juice samples were measured using a mass spectrometer API 4000Q TRAP (AB Sciex, Redwood City, CA, U.S.A.). Initially, the gastric juice was diluted 20– 200-fold using distilled water. Then, 100 µL of the diluted gastric juice was mixed with an internal standard solution (CA-d5 ng/mL in 50% methanol). After mixing the solution by vortexing for 3 s, 200 µL acetonitrile was added, and the mixture was centrifuged at 20,000 × g for 2 min. Subsequently, 20 µL of the diluted supernatant, which was diluted with 180 µL of 20 mM ammonium acetate, was injected for analysis. Standard components of Sigma-Aldrich C9377, G0759, C1129, T4009, L6250, D2510 were used to measure the cholic acid (CA), chenodeoxycholic acid (CDCA), taurocholic acid (TCA), glycochenodexoycholic acid (GCDCA), lithocholic acid (LCA), and deoxycholic acid (DCA), respectively (Sigma-Aldrich Co. LLC, St.Louis, USA). LC–MS/MS data for each bile acid were analyzed using Analyst software version 1.6.3 (AB Sciex Pte. Ltd. Redwood City, CA, USA).

### Diagnosis of *H. Pylori* infection and histology

Diagnosis of *H. pylori* infection was confirmed if any of the four diagnostic tests (rapid urease test, histologic results, *H. pylori* polymerase chain reaction (PCR), and [^13^C]-urea breath test) yielded a positive result. Based on the Vienna classification system, an expert pathologist (CYD) evaluated all biopsy and resected specimens for background histology and tumor histology [10]. 

### Fluid microbiota analysis

DNA was extracted from each fluid sample using the QIAamp® PowerFecal® DNA kit (QIAGEN #12830-50, Hilden, Germany). V3-4 region of the 16 S rRNA gene was PCR amplified using the forward primer (341 F 5′-TCGTCGGCAGCGTCAGATGTGTATAAGAGACAGGTGCCAGCMGCCGCGGTAA-3′) and the reverse primer (806R 5′-GTCTCGTGGGCTCGGAGATGTGTATAAGAGACAGGGACTACHVGGGTWTCTAAT-3′). A MiSeq library was prepared using two-step PCR and sequenced by MiSeq, according to the manufacturer’s instructions (Macrogen Inc., Seoul, South Korea). Sequence data were analyzed according to Miseq S.O.P. (https://mothur.org/wiki/miseq_sop/) using Mothur software [11]. Briefly, paired reads were assembled using make.contigs Mothur subroutine, low-quality reads were removed using screen.seqs Mothur subroutine, the reads were aligned in the SILVA [12] database (version 138), and taxonomic classification was performed using RDP trainset version 18 [13]. All samples were rarefied by randomly sampling 10,000 reads from each sample using sub.sample Mothur subroutine. Alpha-diversity indices (i.e., Chao and Shannon) were calculated using summary.groups Mothur subroutine, and beta-diversity was analyzed based on the Bray–Curtis distance and calculated using dist.shared Mothur subroutine. Non-metric multidimensional scaling (NMDS) and analysis of molecular variance (AMOVA) were performed using NMDS and AMOVA Mothur subroutines, and differentially abundant taxa were identified using the lefse Mothur subroutine [14]. Any genus associated with bile acid components (i.e., CA, CDCA, DCA, TCA, GCDCA, and GDCA) was identified based on Spearman’s correlation analysis.

## Results

### Demographic characteristics of the study population

A total of 50 subjects were enrolled in this study. The purposes of the endoscopic procedures varied among subjects, with 10 subjects undergoing endoscopic surveillance or evaluation of dyspepsia, 21 subjects undergoing endoscopic resection for low-grade dysplasia (LGD), and 19 subjects undergoing endoscopic resection for early gastric cancer (EGC).

The mean age of the entire subjects was 65.0 ± 13.0 years, and 68.0% (34/50) of the subjects were male. An *H. pylori* infection was detected in 46.0% (23/50) of the subjects. Among the subjects, 19 subjects had an endoscopic BR grade 0, 22 had an endoscopic BR grade 1, and 9 had an endoscopic BR grade 2. No significant differences were observed in sex, hypertension, diabetes, *H. pylori* status, main histologic findings, and histologic background status of intestinal metaplasia among the three endoscopic BR grading groups (Table 1).

**Table 1:** Baseline characteristics according to endoscopic bile reflux grading

Parameters	Grade 0 (*n* = 19)	Grade 1 (*n* = 22)	Grade 2 (*n* = 9)	*P*-value
Age, yrs. median (range)	68.0 (27–82)	67.5 (42–81)	62.0 (24–85)	0.663
Male, n (%)	10 (52.6)	17 (77.3)	7 (77.8)	0.189
Hypertension, n (%)	3 (15.8)	4 (18.2)	3 (33.3)	0.534
Diabetes, n (%)	3 (15.8)	3 (13.6)	3 (33.3)	0.411
*H. pylori* infections, n (%)	5 (26.3)	13 (59.1)	5 (55.6)	0.090
Background histologic manifestation of atrophic gastritis with intestinal metaplasia, n (%)	13 (68.4)	19 (86.4)	7 (77.8)	0.384
pH of gastric juice, median (interquartile range), among 36 patients	3.1 (1.7–6.9) among 15 patients	6.8 (5.9–7.2) among 16 patients	7.2 (6.6–7.4) among 5 patients	0.062
Main histologic phenotypes, n (%)				0.100
No neoplasm	7 (36.8)	2 (9.1)	1 (11.1)	
Low-grade dysplasia	6 (31.6)	9 (40.9)	6 (66.7)	
Early gastric cancer	6 (31.6)	11 (50.0)	2 (22.2)	

Endoscopic bile reflux grading. Grade 0: no bile reflux; grade 1: light-yellow-clear fluid in the stomach; grade 2: yellowish-green fluid in the stomach

*H. pylori*: *Helicobacter pylori*

### Association between bile acid concentration and endoscopic bile reflux grading

**Fig. 1:**
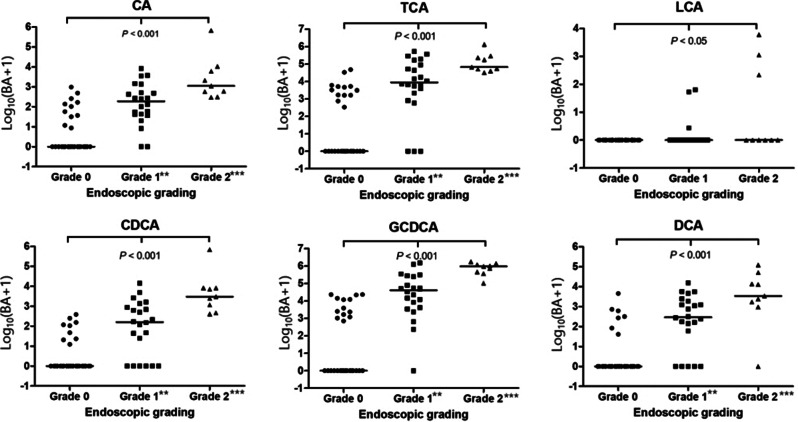
Gastric bile acid concentrations according to the endoscopic bile reflux grading. There was a significant difference in each bile acid level according to the endoscopic bile reflux grading (all *P* < 0.05). There were significant differences in CA, TCA, CDCA, GCDCA, and DCA levels between BR grades 0 and 1 (all *P* < 0.01). In addition, there were significant differences in CA, TCA, CDCA, GCDCA, and DCA levels between BR grades 0 and 2 (all *P* < 0.05). BA: bile acid; CA: cholic acid; TCA: taurocholic acid; LCA: lithocholic acid; CDCA: chenodeoxycholic acid: GCDCA: glycochenodeoxycholic acid; DCA: deoxycholic acid. *P < 0.05, **P < 0.01, and ***P < 0.001 when compared to grade 0

The concentration of conjugated primary bile acids, unconjugated primary bile acids, and secondary bile acids in the gastric juices in the three endoscopic BR grading groups were measured. There was a significant difference in each bile acid level among the three endoscopic BR groups (all *P* < 0.05, Fig. 1). In the post hoc analysis, concentrations of CA, TCA, CDCA, GCDCA, and DCA were significantly higher in the endoscopic BR grade 1 group compared to the endoscopic BR grade 0 group (all *P* < 0.01). Similarly, CA, TCA, CDCA, GCDCA, and DCA concentrations were significantly higher in the endoscopic BR grade 2 group than in the endoscopic BR grade 0 group (all *P* < 0.05).

### Comparison of microbial diversity indices in gastric juice according to the endoscopic bile reflux grading, main histologic diagnosis, *H. Pylori* status, and sex

The refraction curve analysis indicated enough sequence depth (Fig. S2). In addition, all samples showed a coverage higher than 99.5% (data not shown).”

**Fig. 2:**
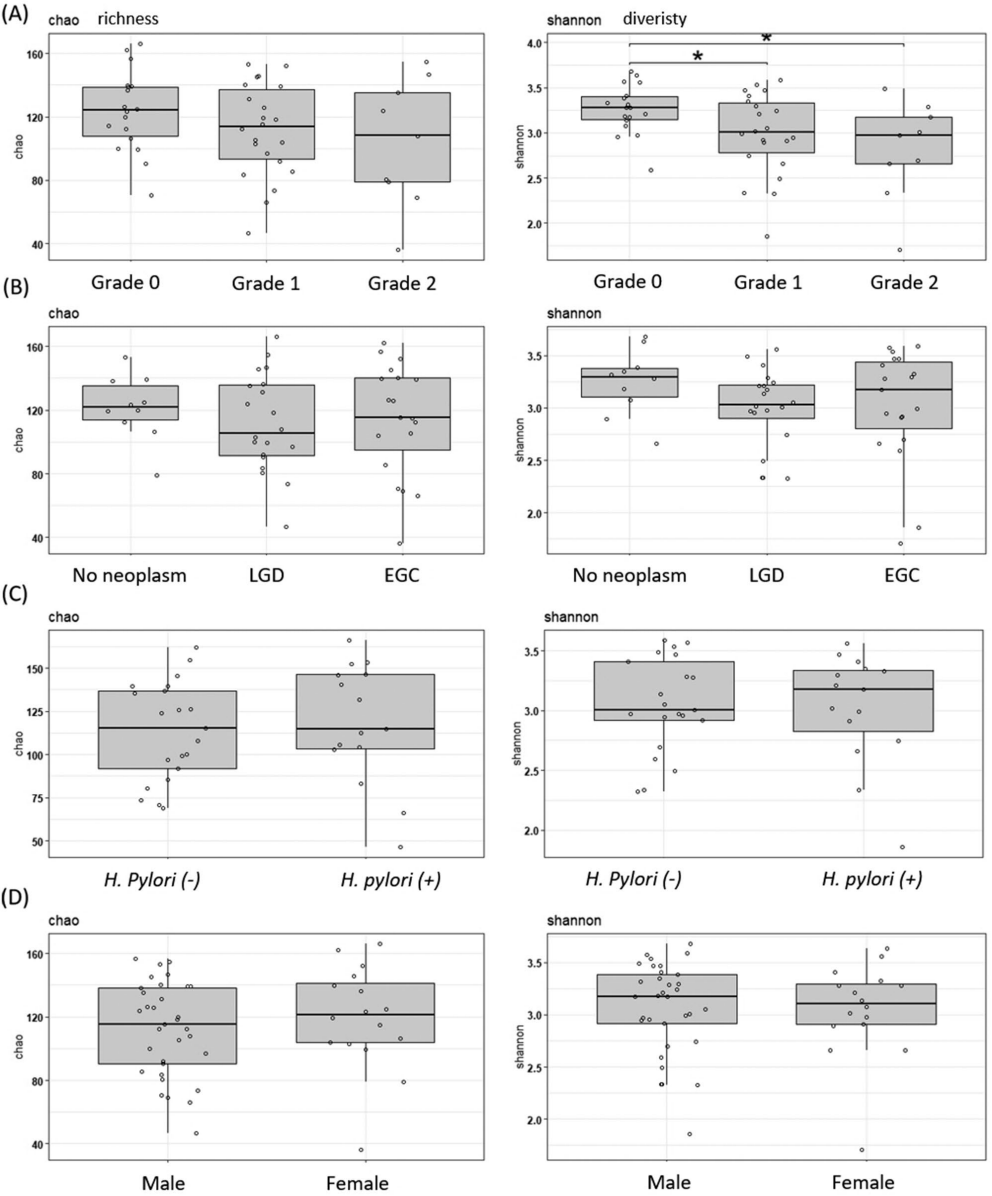
Alpha-diversity comparison based on Chao and Shannon indices according to bile reflux gradings, pathological gradings, *H. pylori* infection, and sex. (**A**) Bile reflux gradings, (**B**) pathological gradings, (**C**) presence/absence of *H. pylori*, and (**D**) sex. Grade 0: no bile reflux; grade 1: light-yellow-clear fluid in the stomach; grade 2, yellowish green fluid in the stomach; LGD, low-grade dysplasia; EGC, early gastric cancer; *H. pylori, Helicobacter pylori*

We evaluated the α-diversity, including species richness (Chao 1 index) and species diversity (Shannon index), in the gastric juice samples based on endoscopic BR grade, main histologic diagnosis, *H. pylori* presence/absence, and sex. Our analysis of the α-diversity demonstrated a significant decrease in microbial diversity as the endoscopic BR grade increased (*P* < 0.05), while no significant differences were observed between groups in the other categories (Fig. 2).

**Fig. 3:**
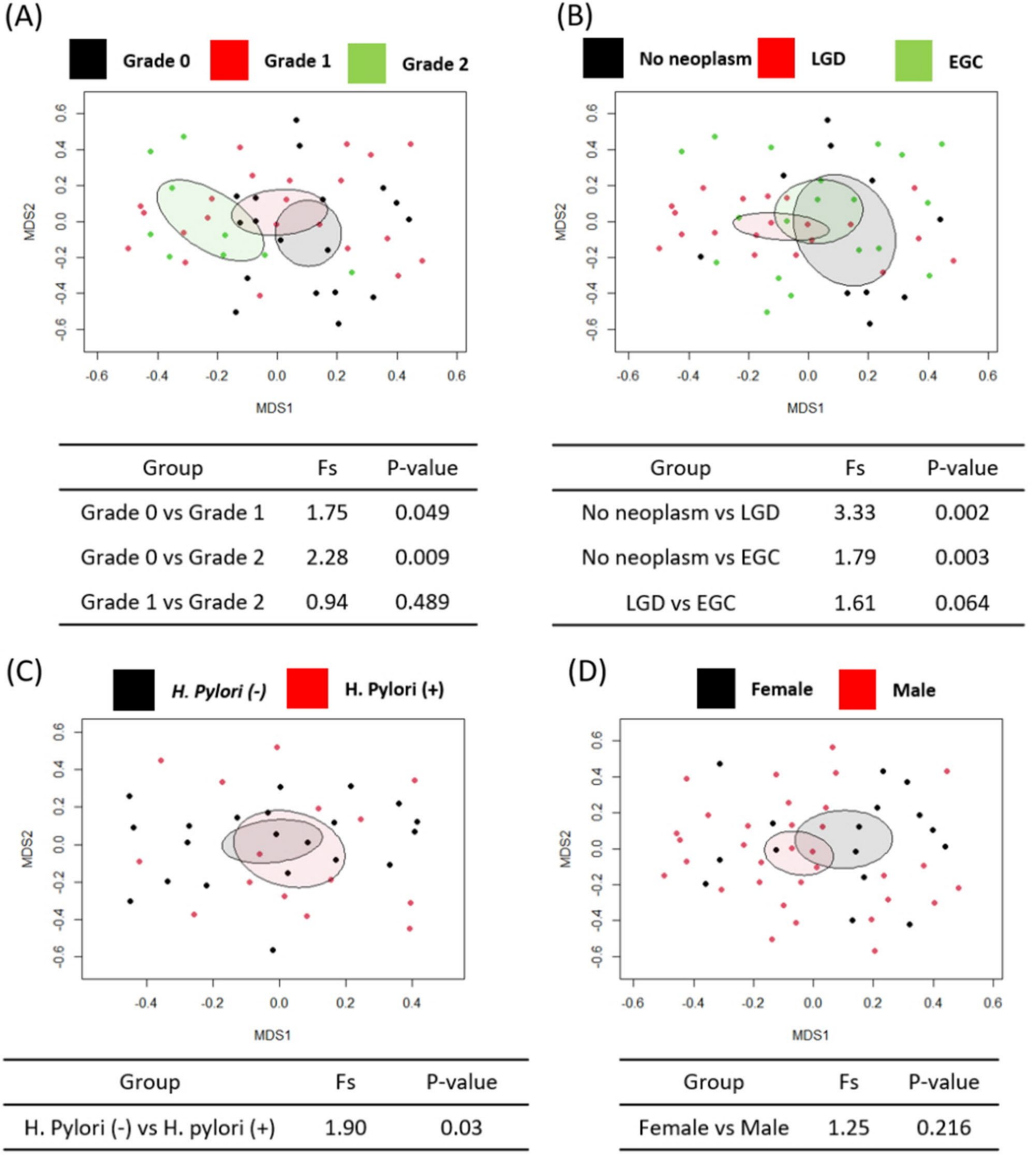
Beta-diversity comparison based on non-multidimensional scaling and AMOVA according to bile reflux gradings, pathological gradings, *H. pylori* infection, and sex. (**A**) Bile reflux gradings, (**B**) Pathologic gradings, (**C**) presence/absence of *H. pylori*, and (**D**) sex. Grade 0, no bile reflux; grade 1, light-yellow-clear fluid in the stomach; grade 2, yellowish green fluid in the stomach; LGD, low-grade dysplasia; EGC, early gastric cancer; *H. pylori, Helicobacter pylori*

To further investigate the differences in the gastric microbiota compositions (β-diversity), we performed NMDS and AMOVA analyses based on the Bray–Curtis dissimilarity values. The β-diversity analysis revealed that bile reflux significantly altered the gastric microbiota composition compared to those without bile reflux, irrespective of the degree of bile reflux (Fig. 3A). Similarly, analyses based on the histological findings showed significant differences in the gastric microbiota between the neoplastic and non-neoplastic groups, although no differences were observed between LGD and EGC (Fig. 3B). Furthermore, the β-diversity analyses showed that the presence of *H. pylori* also resulted in an alteration in the gastric microbiota (*P* < 0.05), while no significant differences were observed according to the subject’s sex (Fig. 3C, D). Since the β-diversity analysis revealed significant differences depending on the presence or absence of BR and neoplasm, we compared the subjects based on the presence/absence of BR with or without neoplasm. Microbial diversity was significantly lowered when neoplasm is present, which can be further reduced when BR is also present, while beta-diversity only showed a significant difference between healthy status (no BR and no neoplasm) and diseased condition (either with BR or neoplasm or both) (Fig. S3).

### Taxonomic composition comparison of the gastric juice microbiota based on endoscopic bile reflux grade and main histologic diagnosis

A comparison of the different endoscopic BR grades revealed that *Firmicutes* and *Bacteroidetes* levels were higher in BR grades 1 and 2, whereas *Proteobacteria* levels were lower compared to BR grade 0. Similarly, patients with LGD and EGC exhibited a higher abundance of *Firmicutes* and lower *Proteobacteria* than the non-neoplastic group (Fig. S4A, B). Our results also showed a significantly higher abundance of *Campylobacterota* in male patients or patients infected with *H. pylori* (Fig. S4C, D).

**Fig. 4:**
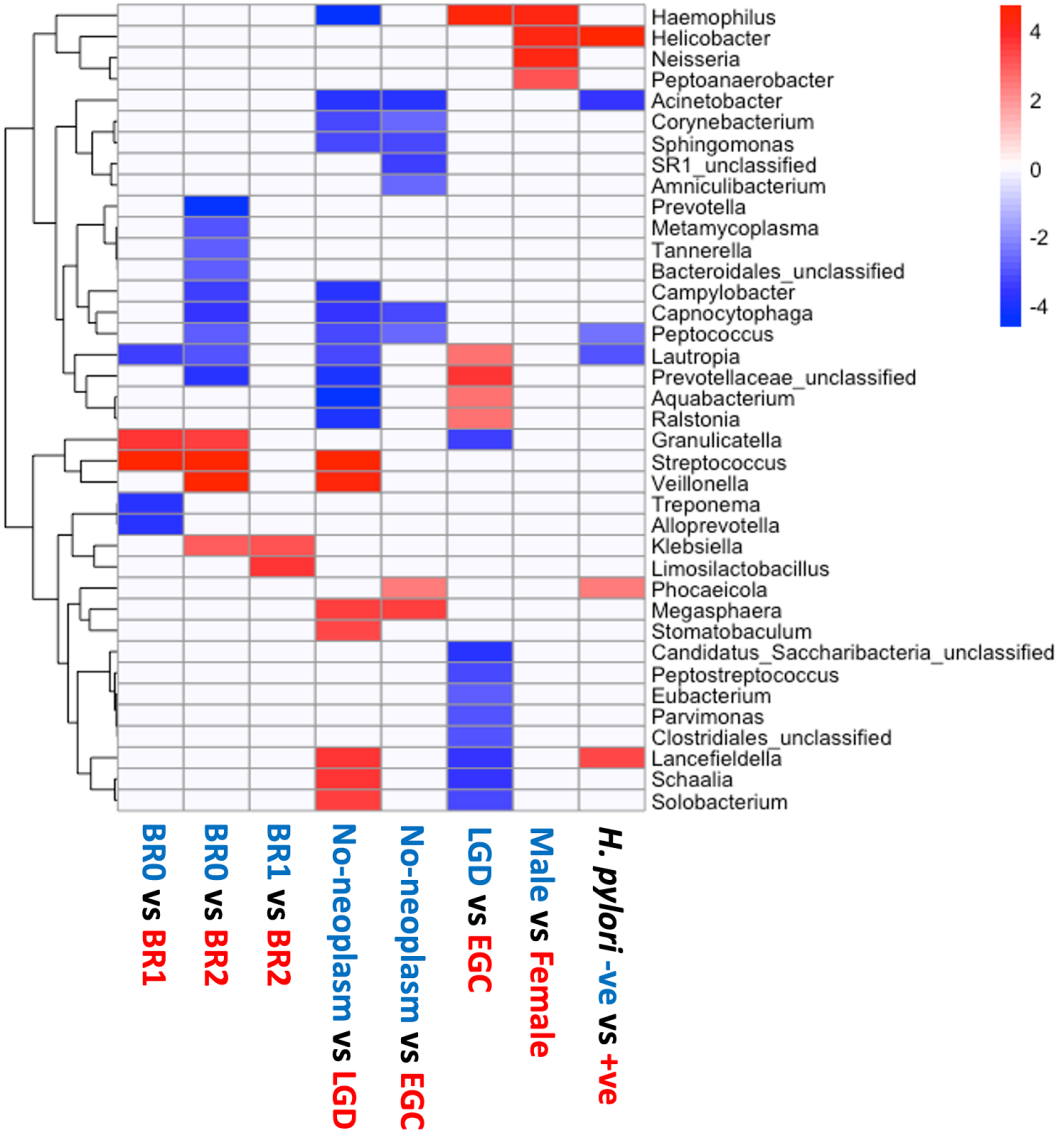
Differential abundance comparison according to bile reflux gradings, pathological gradings, *H. pylori* infection, and sex. LDA effect sizes (> 2.0, *P* < 0.05) were shown in blue and red, indicating each group

At the genus level, the major genera identified included *Streptococcus* and *Prevotella*, followed by *Fusobacterium*, *Haemophilus*, and *Neisseria* (Fig. S5). LefSe analysis was conducted at the genus level to identify genera with significant differential abundance by comparing each category (endoscopic BR grading, main histologic finding, presence of *H. pylori*, and sex) (Fig. 4). Our results showed that *Streptococcus* and *Granulicatella* were significantly enriched in the gastric juices from subjects with BR grades 1 and 2 compared to those with BR grade 0. Conversely, *Lautropia* was significantly lower in the gastric juices from subjects with BR grades 1 and 2. Furthermore, *Klebsiella* and *Limosilactobacillus* were significantly more abundant in BR grade 2 than in BR grade 1. Conversely, different genera were identified from the comparative analyses between pathologic grades. Megasphaera was found to be significantly abundant in both LGD and EGC compared to patients with no neoplasm. Five genera (*Capnocytophaga, Peptococcus, Sphingomonas, Corynebacterium*, and *Acinetobacter*) were significantly higher in the patients with no neoplasm. In addition, we observed four genera that were significantly more abundant in female patients, which included *Helicobacter*. The abundance of *Lancefieldella* and *Phocaecola* was significantly higher in patients with *H. pylori*, whereas *Acinetobacter*, *Peptococcus*, and *Lautropia* were less abundant.

### Genera associated with bile acid components

**Fig. 5:**
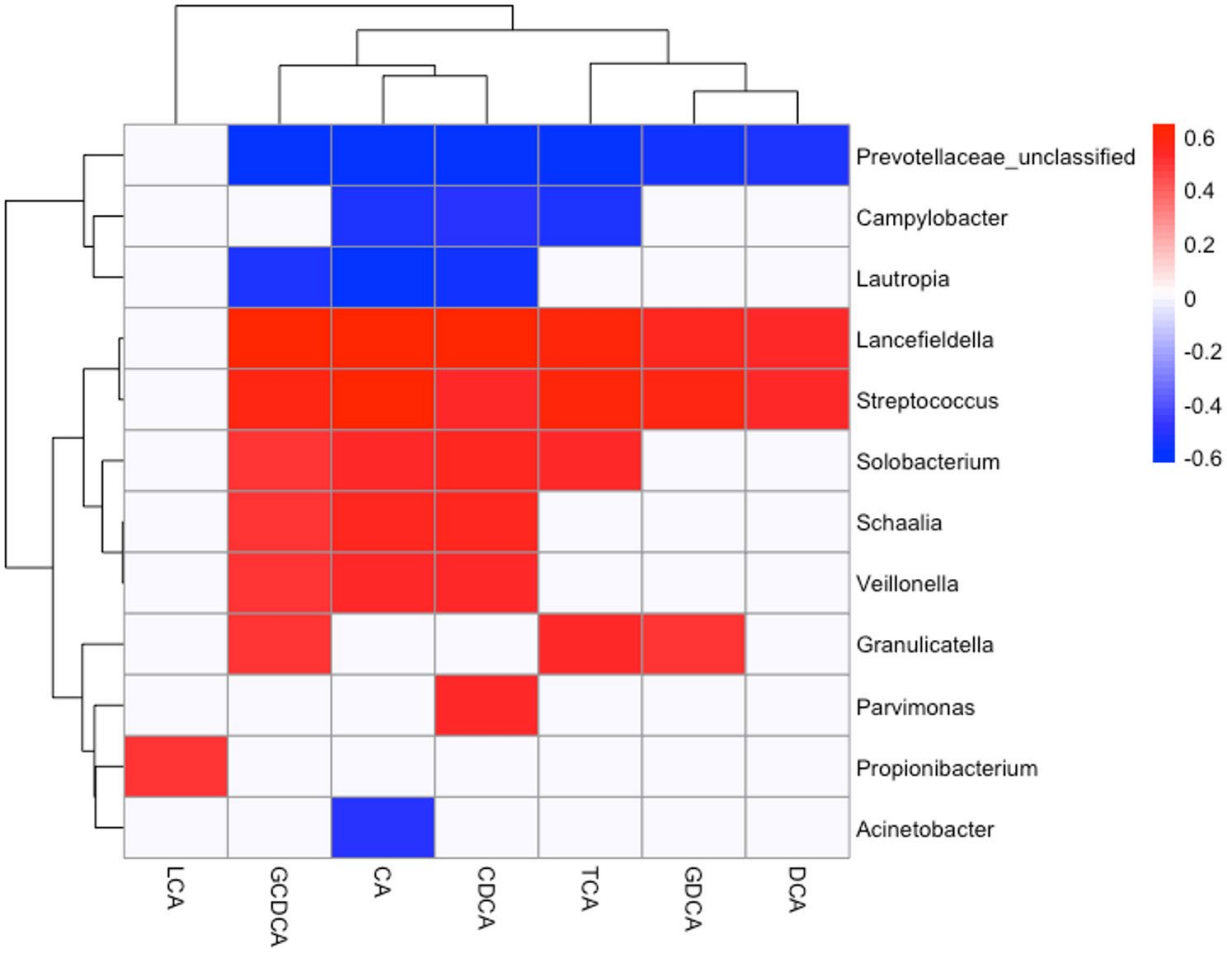
Spearman’s correlation between the abundance of genera and clinical parameters (*P* < 0.05, correlation > 0.5 or < -0.5). CA: cholic acid; TCA: taurocholic acid; LCA: lithocholic acid; CDCA: chenodeoxycholic acid; GCDCA: glycochenodeoxycholic acid; DCA: deoxycholic acid

Spearman’s correlation analysis was performed to assess the relationship between the abundance of each genus and the bile acid components (CA, CDCA, DCA, TCA, LCA, GCDCA, and GDCA). Genera with strong correlation (higher than 0.5 or lower than − 0.5) with significance (*P* < 0.05) are summarized in Fig. 5. The abundance of *Campylobacter* and *Lautorpia* was negatively correlated with three bile acid components, whereas the abundance of *Streptococcus* and *Lancefielfdella* was positively correlated to every bile acid component except for LCA. Moreover, *Granulicatella, Solobacterium*, *Schaalia*, and *Veillonella* positively correlated to many bile acid components. While only one genus, *Propionibacterium*, positively correlated with LCA, the other bile acid components significantly correlated with several genera.

## Discussion

This study demonstrated significant differences in the microbial α-diversity in gastric juice, which were dependent on the BR grade in the stomach. However, there were no significant differences in the microbial α-diversity when comparing the histologic phenotypes, presence of *H. pylori*, and subject’s sex.

Previous studies have shown that the microbiota of gastric mucosa differed from those of gastric juice regarding α-diversity and composition [15, 16]. Sun et al. demonstrated that the α-diversity by Shannon index was higher in the gastric juice than in the gastric mucosa, while using the Chao 1 index revealed no significant differences in the microbiota richness between the two samples [16]. In a study by He, the microbial α-diversity was lower in the gastric mucosa than in the gastric juice. An *H. pylori* infection reduced the α-diversity in the gastric mucosa samples, yet there was no difference in the gastric juice samples following an *H. pylori* infection. However, both an *H. pylori* infection and histologic stages affected the microbial composition in each sample [15]. In this study, we used gastric juices to analyze the microbial composition and reaffirmed the data from previous studies showing that an *H. pylori* infection affects the composition in gastric juice microbiota but not the α-diversity. The temporary presence of bacteria in the gastric juice for a short or unknown period does not penetrate the gastric mucosa or thick mucus layer but creates a gut microbiota environment that differs from that of the gastric mucosa. These results suggest that only procuring a mucosal sample in gastric microbial studies can lead to underestimating the actual effects of microorganisms.

Among many factors affecting the gastric microbiota [7, 17, 18], the process of bile reflux into the stomach may be important for shaping the gastric environment by changing the gastric acidity and GI motility and damaging the gastric mucosa. However, data on the association between BR and gastric microbiota are often limited, and even if reported, the data were only usually generated from gastric mucosal samples. Indeed, Huang et al. reported that chronic gastritis patients with no bile reflux demonstrated a significantly lower microbial richness in mucosal microbiota than those with bile reflux [19]. In contrast, Yang et al. showed no difference in the Chao-1 or Shannon indices regardless of the grades of bile reflux among the mucosal microbiota in patients with chronic gastritis [20]. Since the effect of BR could vary between gastric juice and mucosa, the effects of BR on gastric juice were investigated in subjects with various histological phenotypes. The current study demonstrated that the α-diversity in the gastric juice microbiota was significantly lower in the patients with BR than those without BR. This may be due to the antibiotic effect of bile acid, which increases membrane disruption and leakage of cellular contents and induces DNA damage, protein misfolding, and oxidative stress [21]. The bile acid antimicrobial effect is concentration-dependent, while its sensitivity varies depending on the bacteria’s characteristics, such as efflux pumps, cell wall modification, and an ability to express bile acid exporters or enzymes [22]. Thus, antimicrobial specificity is the potential reason why only diversity and not richness was decreased by BR [21, 23]. 

The microbial compositional comparison using beta-diversity analysis also showed significant differences according to BR, histological phenotype, and *H. pylori* infection, although no significant differences were observed between grades in the same categories (i.e., BR1 vs. BR2; LGD vs. EGC). Our results were inconsistent with previous studies that showed no difference in the beta diversity in the gastric mucosal microbiota between patients with and without BR [19, 20]. As we discussed the inconsistent results for the effect of *H. pylori* infection on α-diversity between mucosal samples and gastric juice samples [19, 20], we speculate that the effects of BR could also differ between gastric juice and mucosal samples. In addition, the previous study showed significant microbial community differences according to the histology [9], whereas our study showed no microbial community difference between EGC and LGD. This may suggest that histology-related microbial changes could be better represented in the mucosa than in the gastric juice.

Previous studies have demonstrated that *Firmicutes*, *Bacteroidetes*, and *Acinetobacter* are dominant phyla in gastric fluid samples, while *Firmicutes* and *Proteobacteria* are most abundant in the gastric mucosal samples [24]. Another study demonstrated that *Firmicutes*, *Proteobacteria*, and *Bacteroidetes* were the most abundant phyla in gastric juice and gastric mucosa despite the slightly different rankings [16]. We also found the gastric juice samples have a relatively higher abundance of the phyla *Firmicutes*, *Bacteroidetes*, and *Proteobacteria* in the present study. In addition, the current study demonstrated a positive correlation between the abundances of *Streptococcus* and *Lancefielfdella* and almost all bile acid components. In addition, the abundances of *Solobacterium*, *Veillonella*, and *Schaalia* were positively correlated with the primary unconjugated bile acids (i.e., CA, CDCA). Previous studies have suggested that bile acid pool size and composition altered gut microbiota composition [25–30]. However, the mechanism through which BAs alter the gut microbiota composition is poorly understood. Previous studies have indicated that BAs have direct antimicrobial effects on gut microbiota through detergent properties and indirect effects on regulating the innate immune defense of the host within the intestine [22, 31, 32], potentially leading to a change in gut microbiota composition. Another plausible mechanism includes the bile salt hydrolase (BSH) of the gut microbiome. BSH enzymes deconjugate bile acids to unconjugated forms, liberating glycine and taurine, which are regarded as nutrient resources for the microbiome. Therefore, gut microbiome composition may differ according to microbial BSH activity [33, 34]. 

Even though gastric mucosal microbiota is potentially more stable and reflects the change in host response through close communication, the change in the gastric mucosal microbiota may have limitations in reflecting the initial change in the intragastric environment following bile acid exposure. In the current study, we have presented the impact of BR on gastric microbiota; nevertheless, we must admit several limitations within this study. Firstly, our analysis was limited only to changes in the microbiota of gastric juice without considering the gastric mucosa. Therefore, it remains uncertain whether the observed effects of BR on the gastric microbiota in gastric juice are consistent in mucosal samples. Secondly, the sample size was relatively small, which may not fully account for the variable gastric environments. Furthermore, there were different pathological grades, even though each group had a similar background histological manifestation of atrophic gastritis with intestinal metaplasia. Therefore, a future study should be performed with long-term follow-up for many patients with simple bile reflux gastritis. Lastly, we failed to obtain medical records (i.e., acid suppressants) from the patients.

In conclusion, microbial changes in gastric juice were associated with BR, and the concentration of refluxed bile positively correlated with the abundance of several specific microbiota. These findings highlight the importance of considering the impact of BR when investigating gastric microbiota. Future studies should aim to comprehensively investigate the effect of BR on both the composition and function of gastric microbiota by using samples from both gastric mucosa and gastric juice.

### Electronic supplementary material

Below is the link to the electronic supplementary material.

Supplementary Material 1

Supplementary Material 2

Supplementary Material 3

Supplementary Material 4

Supplementary Material 5

Supplementary Material 6

## Data Availability

No datasets were generated or analysed during the current study.
