# Supplementary figures and images for: Effect of bile reflux on gastric juice microbiota in patients with different histology phenotypes
Source: Gut Pathog. 2024 May 7;16:26. doi: 10.1186/s13099-024-00619-7 (PMC11077708; doi:10.1186/s13099-024-00619-7)

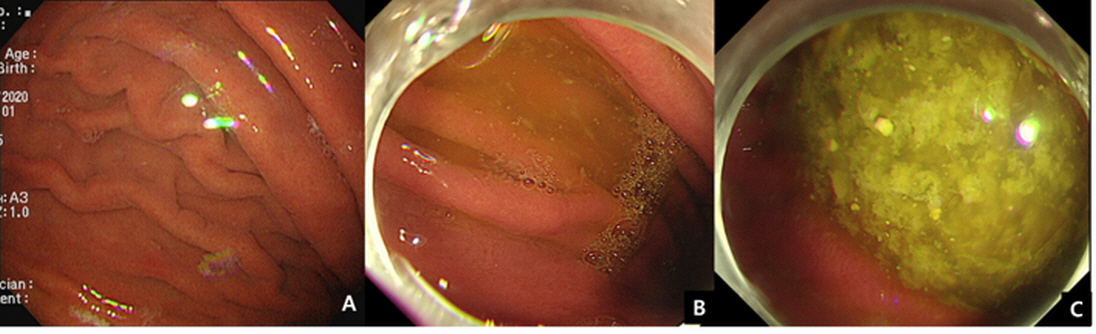

Supplement: Supplementary file 1 — Supplementary Material 1 [file 13099_2024_619_MOESM1_ESM.jpg]

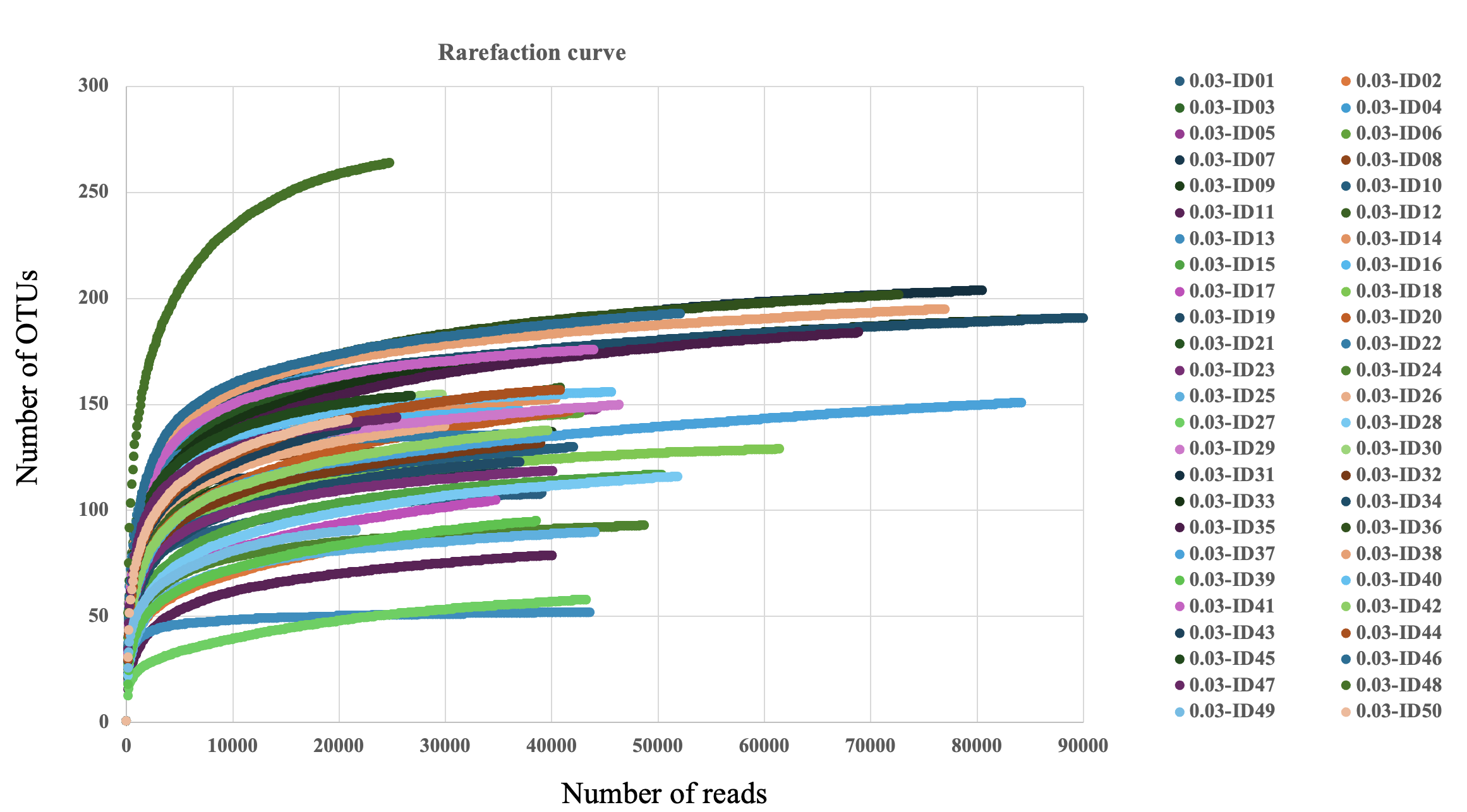

Supplement: Supplementary file 2 — Supplementary Material 2 [file 13099_2024_619_MOESM2_ESM.png]

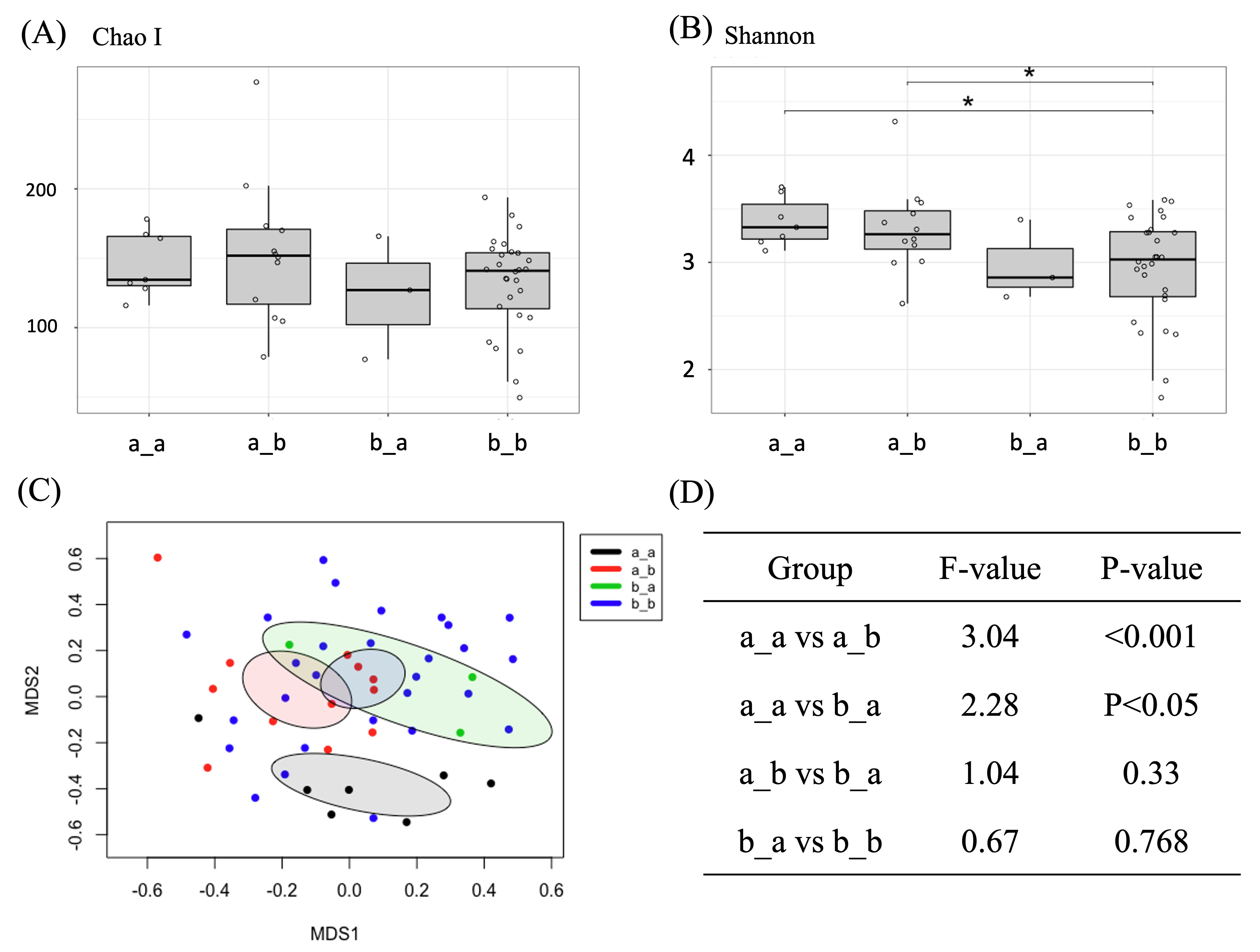

Supplement: Supplementary file 3 — Supplementary Material 3 [file 13099_2024_619_MOESM3_ESM.png]

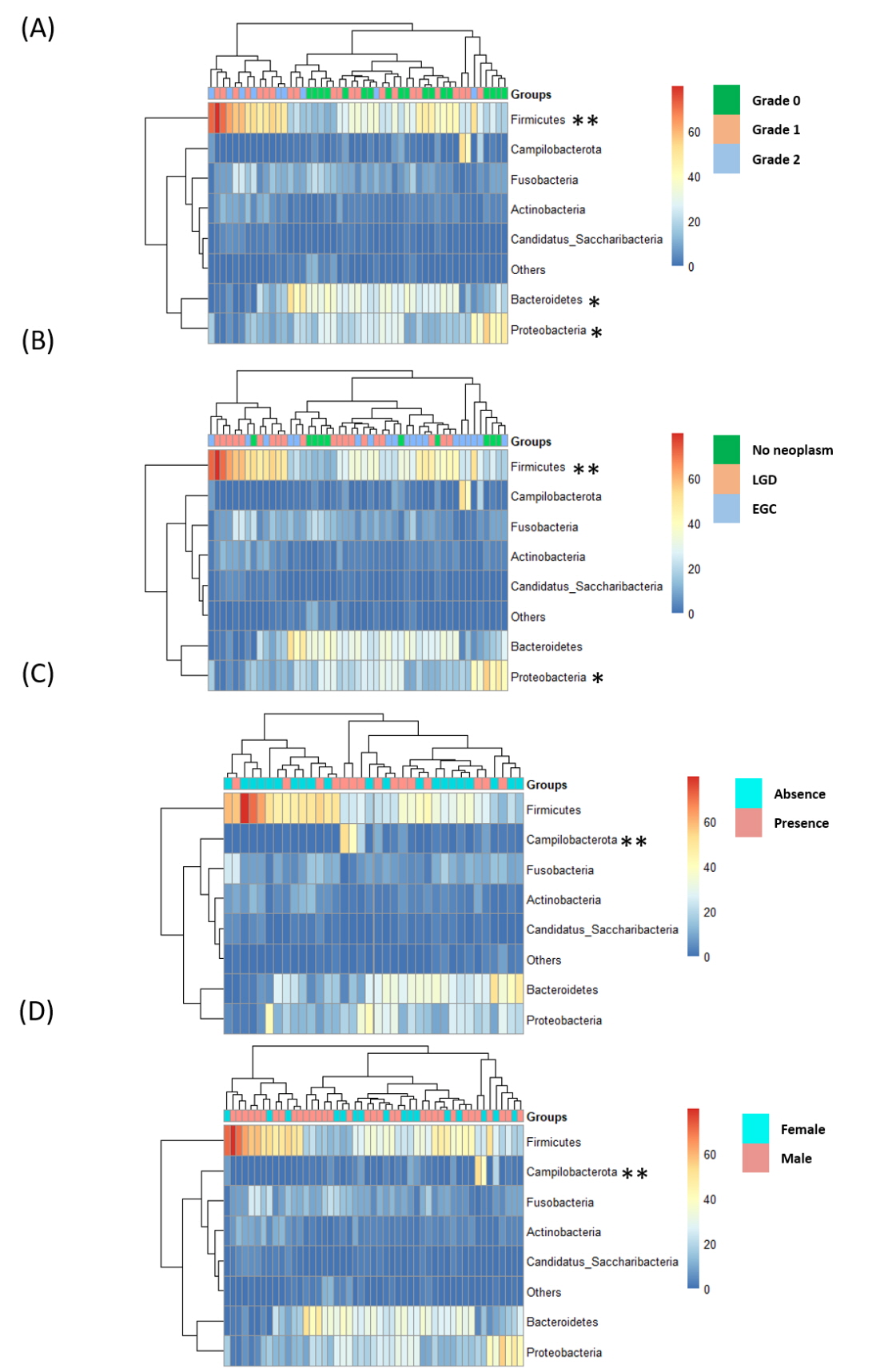

Supplement: Supplementary file 4 — Supplementary Material 4 [file 13099_2024_619_MOESM4_ESM.jpg]

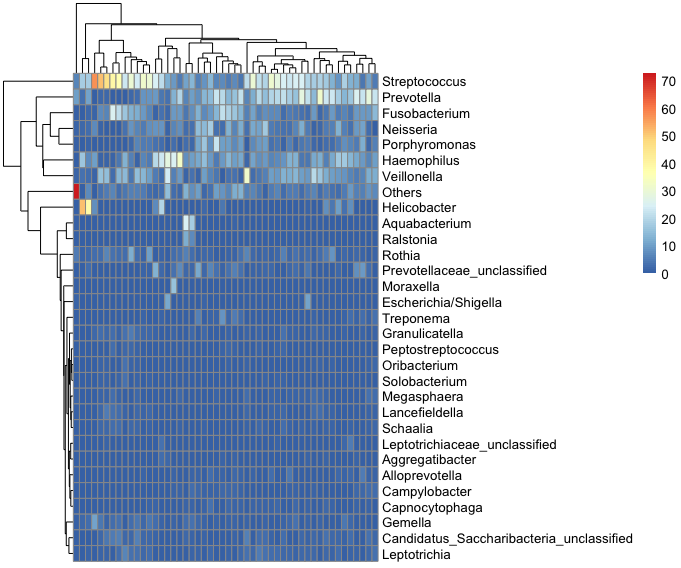

Supplement: Supplementary file 5 — Supplementary Material 5 [file 13099_2024_619_MOESM5_ESM.png]
