# Supplementary material for: Effect of bile reflux on gastric juice microbiota in patients with different histology phenotypes
Source: Gut Pathog. 2024 May 7;16:26. doi: 10.1186/s13099-024-00619-7 (PMC11077708; doi:10.1186/s13099-024-00619-7)
Supplement: Supplementary file 6 — Supplementary Material 6 [file 13099_2024_619_MOESM6_ESM.docx]

**Supplementary figure legends**

**Figure S1. Endoscopic bile reflux grading.** (A) grade 0: no bile reflux, (B) grade 1: light yellow clear fluid in the stomach, (C) grade 2: yellowish green fluid in the stomach.

**Figure S2. Refraction curve analysis.** The refraction curve analysis indicated enough sequence depth. All samples showed a coverage higher than 99.5% (data not shown)."

**Figure S3. Alpha- and beta-diversity comparisons between bile reflux with or without neoplasms**. (A) Chao I species richness; (B) Shannon diversity; (C) Non-metric multidimensional scaling (NMDS); and (D) analysis of molecular variance (AMOVA). a_a, a_b, b_a, and b_b indicate BR 0 with no neoplasm (n = 7), BR 0 with LGD & EGC (n = 12), BR 1 & 2 with no neoplasm (n = 3) , and BR 1 & 2 with LGD & EGC (n = 28).

**Figure S4. Taxonomic composition comparison at the phylum level.** (A) bile reflux gradings, (B) Pathologic gradings, (C) presence/absence of *H. pylori*, and (D) sex. * and ** indicate significantly higher and lower abundances in the control samples in (A) and (B), respectively, whereas for an absence of *H. pylori* and in females in (C) and (D), respectively (*P* < 0.05).

**Figure S5. Taxonomic composition of top 30 abundant genera.**
